# Controllable liquid colour-changing lenses with microfluidic channels for vision protection, camouflage and optical filtering based on soft lithography fabrication

**DOI:** 10.1186/s40064-016-2231-4

**Published:** 2016-05-10

**Authors:** Min Zhang, Songjing Li

**Affiliations:** Department of Fluid Control and Automation, Harbin Institute of Technology, Science Park, No. 2, Yikuang Street Nangang District, Box 3040, Harbin, 150001 China

**Keywords:** Liquid colour-changing lens, Microfluidic channel, Soft lithography, Vision protection, Camouflage, Optical filtering

## Abstract

In this work, liquid colour-changing lenses for vision protection, camouflage and optical filtering are developed by circulating colour liquids through microfluidic channels on the lenses manually. Soft lithography technology is applied to fabricate the silicone liquid colour-changing layers with microfluidic channels on the lenses instead of mechanical machining. To increase the hardness and abrasion resistance of the silicone colour-changing layers on the lenses, proper fabrication parameters such as 6:1 (mass ration) mixing proportion and 100 °C curing temperature for 2 h are approved for better soft lithography process of the lenses. Meanwhile, a new surface treatment for the irreversible bonding of silicone colour-changing layer with optical resin (CR39) substrate lens by using 5 % (volume ratio) 3-Aminopropyltriethoxysilane solution is proposed. Vision protection, camouflage and optical filtering functions of the lenses are investigated with different designs of the channels and multi-layer structures. Each application can not only well achieve their functional demands, but also shows the advantages of functional flexibility, rapid prototyping and good controllability compared with traditional ways. Besides optometry, some other designs and applications of the lenses are proposed for potential utility in the future.

## Background

Microfluidics technology has been the focus of intense research and development as it promises a multitude of advantages in a number of markets including chemical and biological analysis (Shih et al. 2015; Liberale et al. 2013), drug delivery (Majedi et al. 2013) and medical diagnose (Lee et al. 2014; Ng Alphonsus et al. 2010), such as small sizes, high throughput and low cost of microfluidic systems (Paul et al. 2006). Microfluidic has also revolutionized some aspects of optical area (Tseng et al. 2009; Liu et al. 2012). Lim et al. (2014) reported a microfluidic optical fiber devices composed of microfluidic channels which can be used for sensitive refractive index sensing and biosensing applications. Fuentes-Fernandez et al. (2013) proposed an electrowetting-based variable focus liquid lens used for curvature sensors, which can reduce the overall size of the system without the need of extra moving parts. In recent years, a few examples of surface property control (shape, pressure etc.) of materials through microfluidic combined with optics were reported (Iimura et al. 2015). Roy and Ghatak (2014) designed an adaptable optofluidic aspherical lenses by using elastocapillary instability induced by surface tension of a soft rubbery layer with microfluidic channels. Chang et al. (2009) presented a flexible material of controlled shape and stiffness embedded with microchannel networks. When the channels were filled with photoresist, deformed and exposed to UV light, the photoresist inside the channels was solidified, locking in the programmed shape of the materials. However, the reports on the applications of colour control in optometry by using microfluidic are few.

In our daily life, wearing colour-changing sunglasses has become popular way for vision protection and aesthetic increasing. The traditional colour-changing glasses are made of solid photochromic glass containing silver halides (Armistead and Stookey 1964; Tian and Zhang 2012) inside, which can change their molecular construction for colour changing under different light conditions. But these solid photochromic glasses have shortcomings, such as single colour, poor controllability on colouration process and high price.

Camouflage glasses are essential equipments for soldiers or hunters in the wild to blend with the surroundings for self-camouflage. Compared with common used camouflage nets, they have higher transparency and more flexibility for faces concealing. Camouflage technology in the animal field has been extensively studied and increasingly used by human in previous literatures (Surmacki et al. 2013; Watson et al. 2014; Dimitrova and Merilaita 2014). Kang et al. (2015) presented experiments and discussions about the concealing mechanisms of moths during behavioral choice of a resting position, which told us that some species reinforce their crypticity in terms of both background matching and disruptive colouration to improve camouflage against natural predators. Yu et al. (2014) conducted an adaptive optoelectronic camouflage systems with designs inspired by cephalopod skins, which provided critical capabilities in distributed sensing and actuation for mimicking biological colour tuning. These camouflage ways are fine for body-concealing, but impossible for optometry, because optical transparency is not considered in these systems. Moreover, complex principle and structure hard to mimic are mostly involved for achieving near perfect camouflage result in their systems. Morin et al. (2012) reported a soft machine with microfluidic networks which could realize camouflage/display of the body surface by pumping different colour liquids into the channels. But little attention was paid to the characteristics and applications of microfluidic in optometry. Meantime, the fabrication process of this soft machine was based on conventional soft lithography way (McDonald et al. 2000; Becker and Gaertner 2008), of which the key fabrication parameters and bonding method are weak for the manufacturing of camouflage glasses based on the optical resin material (CR39).

Optical filters have been widely used for photo taking to obtain different photography effects. Traditional optical filters in the market are made of coated glass or plastic. The main drawbacks of these coated filters are singleness of filter function and complexity of making process (Taichung and Hsinchu 2004; Moon et al. 2008). Furthermore, the uniformity of the coating, especially on the edges, remains a critical technical issue for traditional manufacture techniques (Yoon and Lee 2010; Yang et al. 2015).

Here, we fabricate liquid colour-changing lenses with microfluidic channels based on soft lithography with proper fabrication parameters. For irreversible bonding of silicone colour-changing layer with optical resin lens (CR39), a new surface treatment way by using 5 % (volume ratio) 3-Aminopropyltriethoxysilane (APTES) solution is investigated and validated. By carefully designing of the channels and controllably circulating proper colour liquids through the channels, the liquid colour-changing lenses can be used for vision protection, camouflage and optical filtering. Compare with conventional ways, these applications of the lenses can not only well achieve the functional demands, but they also show the advantages of simple principle, flexible function and good controllability. Meantime, the liquids filled in a cavity structure usually used in previously published or commercialized adjustable spectacles (Ren and Wu 2005; Santiago-Alvarado et al. 2013; Zhao et al. 2015) may be easily affected by gravity, which will result in incompletely replacing from the cavity, the liquid colour-changing lenses with the design of microfluidic channels adopted in this paper are the key to this problem, which can realize reliable colour liquids circulation.

In this paper, the liquid colour-changing lenses with microfluidic channels are presented and fabricated based on soft lithography. Proper fabrication parameters and surface treatment way are investigated. Different shapes and dimensions of the channels are designed and applied for vision protection, camouflage and optical filtering.

## Working principle and fabrication

**Fig. 1:**
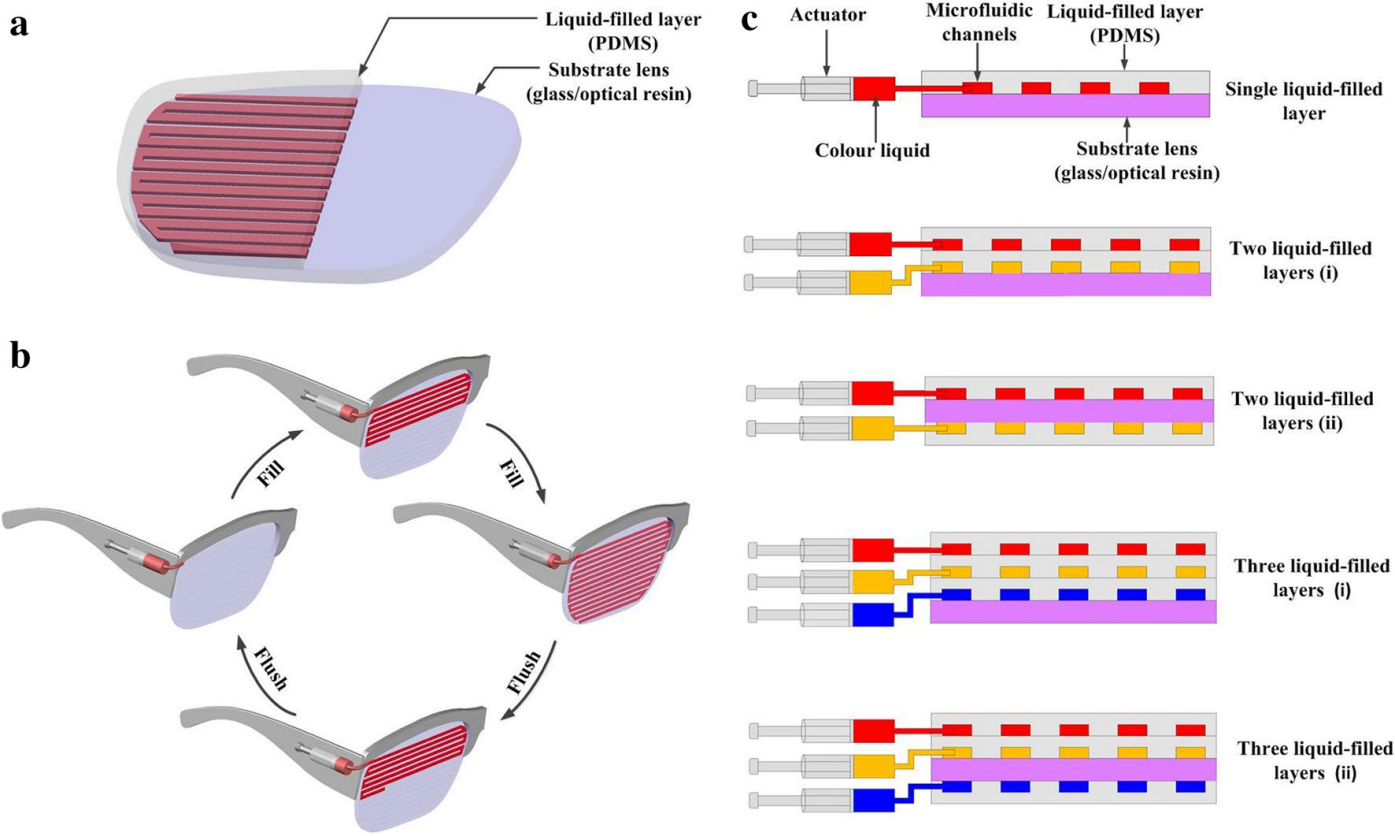
**a** The structure of the liquid colour-changing lens with microfluidic channels. **b** The colour liquid circulation in microfluidic channels by a manual actuator. **c** Various designs of single or multiple liquid colour-changing layers

The liquid colour-changing lens is basically composed of a liquid colour-changing layer with microfluidic channels and a substrate lens (glass/optical resin), as shown in Fig. 1a. The liquid colour-changing layer with microfluidic channels is bonded to the substrate lens to form closed microfluidic channels. Colour liquids will be circulated into the channels and turn the lens into a colour as the users wishes, as well as circulated out of the channels and make the lens become transparent when needed. Compared with conventional colour-changing lens made of solid photochromic materials, actuators for controlling the liquids circulation in the channels on the lens will be needed. The actuators can be manually controlled without batteries. A simple manual actuator with a piston moved forward and backward by fingers is adopted in this paper, as shown in Fig. 1b. The size of the piston is small enough to circulate the amount of liquids through the lens.

The shape and size of the channels can be diversified designed for different functions. In this paper, when the channels are carefully designed and proper liquids are selected for vision protection, diversity of colours, high transparency and effective ultraviolet resistance can be realized without bringing any side effect to human vision. When disruptive channels are designed and appropriate colour liquids are filled to match the background, good camouflage effects of the lens can be achieved. If the liquid colour-changing lens is used as optical filter, different wavelengths of monochromatic light can be absorbed by the colour liquids selected and various photography effects can be obtained. Moreover, in order to realize gradient overlay effect of colours and satisfy personalized requirements of wearers, two or more liquid colour-changing layers filled with different colour liquids are designed and fabricated, as shown in Fig. 1c.

In this research, the liquid colour-changing layer of the lens is made of polydimethylsiloxane (Duffy et al. 1998; McDonald and Whitesides 2002) (PDMS) silicone which is less fragile, less expensive and has good optical transparency. More importantly, it is very convenient to make microfluidic devices with PDMS silicone by using soft lithography technology for fast prototyping and without involving any mechanical manufacturing.

**Fig. 2:**
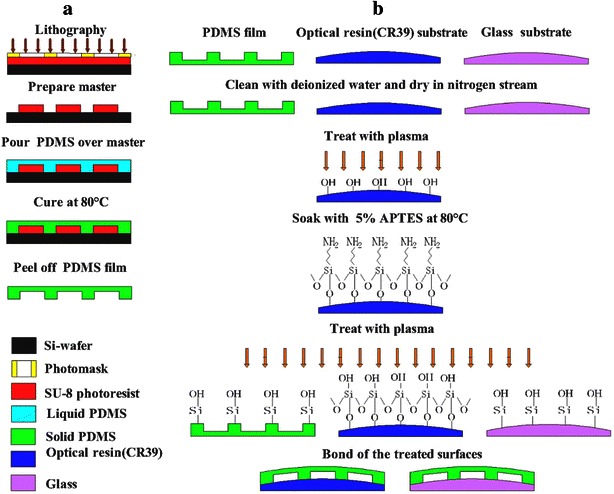
Fabrication of the liquid colour-changing lens with microfluidic channels. **a** Fabrication of the PDMS film with microfluidic channels. **b** Bonding process of the PDMS film with different substrate lenses (glass/optical resin CR39)

Soft lithography process has been widely used in the fabrication of microfluidic systems nowadays (McDonald et al. 2000; Becker and Gaertner 2008). Most often, 10:1 mixture of PDMS polymer and curing agent, and 80 °C curing temperature for 40 min are used in soft lithography process. However, in order to improve the hardness of the PDMS film and help increasing the abrasion resistance of the lens film, more curing agent, higher curing temperature and longer curing time are tried in this research. The fabrication process of the PDMS film with microfluidic channels is shown in Fig. 2. Soft lithography starts with the production of a master containing the channel patterns based on fast prototyping (Qin et al. 1996). In this technique, the channel designs generated by CAD program is printed on a transparent film (Fujifilm Company, Japan) by using a film printer (EPSON Limited Company, Japan) at 2400 dpi (or better resolution); this transparent film is used as the mask in photolithography. SU-8 photoresist (Wenchang Chip Technology Limited Company, China) is coated on a silicon wafer (Wenchang Chip Technology Limited Company, China) for a given thickness by using a spin-coater (The Institute of Microelectronic, China). After photolithography, the channel pattern is transferred from the photomask to SU-8 photoresist to be served as the master in soft lithography. A mixture of PDMS polymer and the curing agent (Dow Corning Corporation, USA) is prepared at the ratio of 6:1 in weight. Pour this prepared PDMS mixture onto the master and let stand for 40 min for degassing. Then put the wafer into a vacuum oven (Tianjin Taisite Instrument Limited Company, China) and cure for 2 h at 100 °C. After curing process, peel off the PDMS film from the master and move to the bonding process.

The bonding process of the PDMS film to the substrate lens can be reversible and irreversible. The reversible bonding of a microfluidic system is not tight enough, but the dismantled microfluidic system can be reused after cleaning. The irreversible bonding is sufficiently strong to withstand a high supply pressure, but the internal cleaning of the channels would be more difficult. In this paper, the latter is adopted for the bonding of PDMS film to optical resin lens (CR39). Plasma treatment has been validated and used extensively for irreversible bonding of PDMS to PDMS/glass substrate in the literatures before (Thuillier and Malek 2005; Eddings Mark et al. 2008; Hemmil et al. 2012). But for CR39 substrate lens, only plasma treatment is not strong enough to form irreversible bonding. In this paper, the irreversibly bonding of PDMS with CR39 substrate lens is successfully realized by surface modification with 5 % (volume ratio) 3-Aminopropyltriethoxysilane (APTES) solution, as shown in Fig. 2b. A 1 mm thick PDMS film and a commercial 2 mm thick CR39 optical lens (Mingyue Optical Limited Company, China) are rinsed with deionized water and dried in nitrogen stream (Yuanye Biotechnology Limited Company, China). For better silylation effect, the latter lens is activated in a plasma cleaner (Mingheng Technology Limited Company, China) for 40 s with the power of 80 W and air flow of 400 mL/min. The CR39 optical lens is then submerged in 5 % APTES solution (Yuanye Biotechnology Limited Company, China) at 80 °C for 30 min for surface silylation, which can introduce Si-containing groups () on the lens surface. Then both the PDMS and silylated CR39 substrate lens are subjected to another plasma treatment (80 W, 400 mL/min) for 40 s for the formation of hydrophilic groups (Si–OH) on their surfaces. Immediately after the treatment, bond the two treated surfaces together and strong Si–O–Si covalent bonds will be formed between the bonding surfaces. Baking the bonding structure in a vacuum oven at 60 °C for 12 h to make sure of that the bonding is strong enough. In order to validate the effectiveness of the surface treatment, the bonding strength between PDMS and CR39 substrate lens is examined by tensile tester (Handpi Instrument Limited Company, China). For APTES-assisted sealing, the bonding strength of the structure is 1060 kPa, which is much higher than 650 kPa obtained based on plasma treatment alone.

**Fig. 3:**
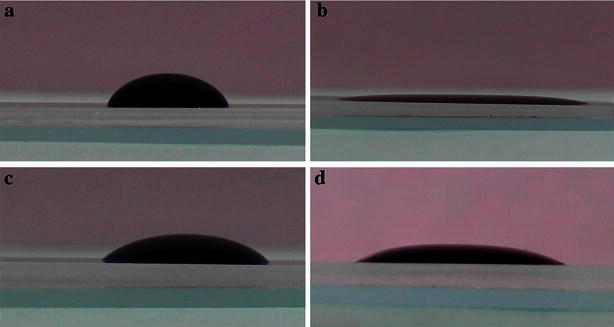
Advancing contact angles of CR39 after passing different treatments. **a** Before treating. **b** After plasma treating. **c** After plasma-APTES treating. **d** After plasma-APTES-plasma treating

To verify the changing process of the surface properties, the contact angles of the CR39 sheets (60 × 30 × 2 mm) after passing different treating processes are measured and compared with each other. 0.1 mL volume of dyed water is added on the surface of the CR39 sheet and the photographs are taken by digital camera (Canon Incorporation, Japan) within 2 min after treating for contact angle measurements, as shown in Fig. 3. Under each experimental condition, the photographs are taken for five times and the average value of the advancing contact angle is calculated. Before treating, the advancing contact angle of CR39 sheet is around 60° (Fig. 3a). After treating by air plasma, it is only around 10° (Fig. 3b), due to the formation of high hydrophilic hydroxy groups (–OH) on the treating surface. Then it increase to 45° (Fig. 3c) after surface silanization with 5 % APTES solution, because of the presence of the amino groups () with poor hydrophily. After another plasma treating, terminal amino groups () converse to more hydrophilic hydroxy groups (Si–OH), and lower contact angle of 22° (Fig. 3d) is obtained. Therefore, the variation of the advancing contact angles can indicate the groups changing on the treated surface.

## Applications and discussion

With different shapes and designs of microfluidic channels, the liquid colour-changing lenses can be applied for vision protection, camouflage and optical filtering respectively.

### Colour-changing glasses for vision protection

**Fig. 4:**
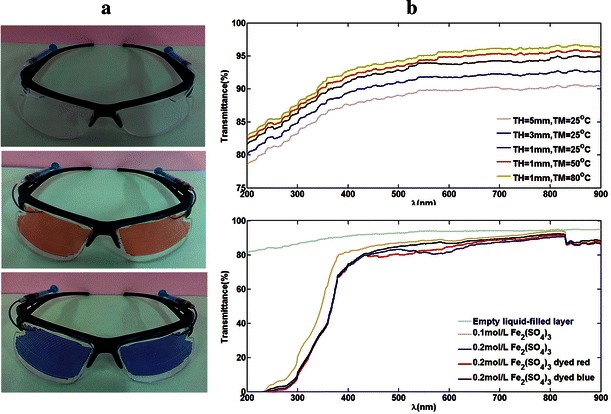
Colour-changing glasses for vision protection. **a** Colouration photographs with or without colour liquids. **b** The transmittance of PDMS films with different thickness at different temperature (*top*) and transmittance of the colour-changing lenses filled with different Fe_2_(SO_4_)_3_ solutions (*bottom*)

Figure 4 shows the colour-changing glasses for vision protection based on the liquid colour-changing lens with microfluidic channels. The width and interval of the microfluidic channels are 0.75 and 0.5 mm respectively. The depth is made to be 0.05 mm. Different colour-changing effects are presented in Fig. 4a. Meantime, the transmittance tests are conducted by using a spectrophotometer (Shimadzu Corporation, Japan). It has self-contained light sources which can produce continuous spectrum with the wavelength ranging from 200 to 900 nm. Through a monochromator, a specific wavelength of monochromatic light can be obtained. The colour-changing lens under test was vertically placed on the sample stage situated behind the monochromator and in front of the optoelectric detectors. The transmitted reference signal and sample signal can be detected and compared by optoelectric detectors. Then the transmittance of the lens can be obtained and appear on the display.

In order to understand the effect of thickness and temperature on the transmittance of PDMS film, different sizes of PDMS films (10 × 5×1 cm, 10 × 5×3 cm and 10 × 5×5 cm) are fabricated and tested. The transmittance of all the PDMS films is over 80 % in different wavelengths of light, especially in visible light, more than 85 % transmittance is achieved, as shown in Fig. 4b (top). Meanwhile, it also indicate that the transmittance of the PDMS film gradually decreases with the increasing of its thickness, and increases with the rising of environment temperature. Generally, PDMS films with different thickness at different temperature can all meet the optometry demands with the satisfying transmittance.

Capable of absorbing the ultraviolet radiation in the sunlight, different concentrations of Fe_2_(SO_4_)_3_ solutions (Yuanye Biotechnology Limited Company, China) are filled in the microfluidic channels on the lens for experimental tests. The transmittance decreases significantly in ultraviolet region but keep above 80 % in visible light region, as shown in Fig. 4b (bottom). At a wavelength of 250 nm, only 4.29 % ultraviolet light can pass through when the lens is filled with 0.1 mol/L Fe_2_(SO_4_)_3_ solution, and down to 1.84 % when 0.2 mol/L Fe_2_(SO_4_)_3_ is applied. Therefore, this liquid colour-changing lenses demonstrate good functions of anti-ultraviolet rays and transmission of light. Meantime, in order to realize various colour-changing effects, the Fe_2_(SO_4_)_3_ solutions dyed red and blue are used to change the exterior colour of the lens. Little impact on transmittance of the lens is found after dyeing (Fig. 4b).

Some other personal designs of microfluidic channels and multilayer channels can also be easily achieved to meet different fashion desires of the wearers. Moreover, different optometry functions can be implemented when the lenses are filled with different solutions.

### Camouflage glasses

**Fig. 5:**
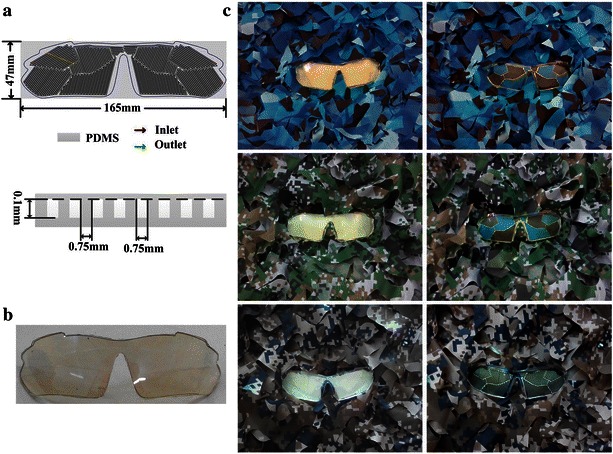
Camouflage glasses. **a** Design of the microfluidic channels on the lens (*top*) and cross-sectional schematic of the region indicated by the *dotted yellow line* (*bottom*). **b** Colse-up image of the camouflage lens without camouflage effect. **c** Images of the lenses with and without camouflage in different backgrounds

By designing disruptive channels to break up the appearance of the lens body and filling in proper colour liquids, the liquid colour-changing lens can realize background matching and camouflage functions, as shown in Fig. 5. Figure 5a is the designing of the microfluidic channels on the lenses. The width and interval of the channels are 0.75 and 0.75 mm respectively, the depth is 0.1 mm. Figure 5b is the image of the camouflage lens without camouflage effect which shows good transparency. Figure 5c shows the images of the lenses with and without camouflage in three different backgrounds.

**Fig. 6:**
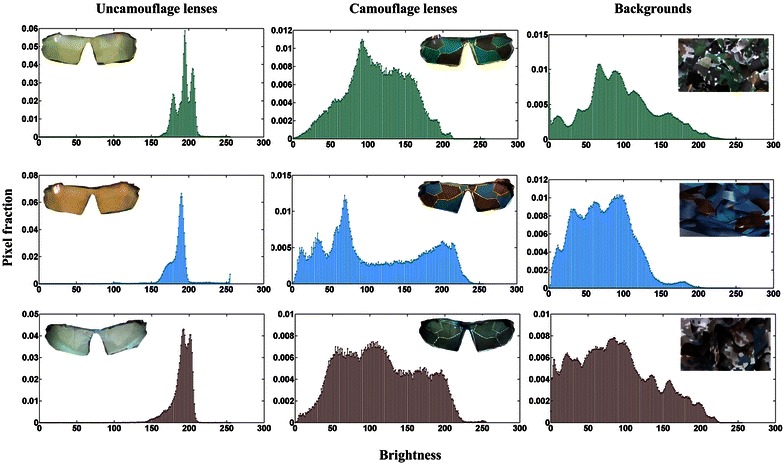
Stacked histograms showing the distribution of gray-scale intensities (brightness) generated by image analysis of isolated uncamouflage lenses, isolated camouflage lenses and their backgrounds

To show the effectiveness of the camouflage, basic image analysis are performed by focusing on brightness between the lenses and their backgrounds. The images of uncamouflage lenses, camouflage lenses and their backgrounds to be analyzed are prepared. A manually drawn masking layer to the region with a lens is applied to isolate the lens from the background by Photoshop software. The isolated uncamouflage lenses, isolated camouflage lenses and their backgrounds are converted to grayscale with pixel brightness represented by an 8-bit number between 0 (black) and 255 (white). The pixel brightness histograms are computed for these images utilizing MATLAB program, as shown in Fig. 6. For isolated uncamouflage lenses (Fig. 6 left), the brightness of the most pixels is concentrated in 150–210. For isolated camouflage lenses (Fig. 6 middle) and their backgrounds (Fig. 6 right), they are all distributed in 0–220 and have similar arrangements. Therefore, it is demonstrated that the mean brightness of the camouflage lenses are much closer to those of their backgrounds than those of uncamouflage lenses.

**Fig. 7:**
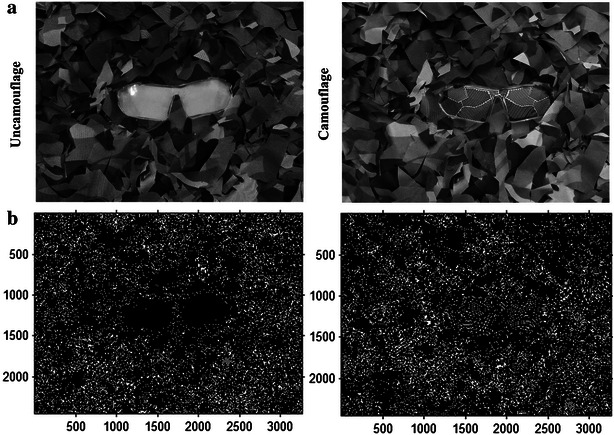
“Canny” edge-finding algorithm analysis. **a** Grayscale images of camouflage lens and uncamouflage lens. **b** “Canny” edge-finding algorithm analysis diagrams for the images in Fig. 6a

For further analysis of the camouflage effects, “Canny” edge-finding algorithm is applied to one group of images [Fig. 5c (top)] for edge comparison. The images of uncamouflage lens and camouflage lens to be analyzed are converted to grayscale by Photoshop software, as shown in Fig. 7a. “Canny” edge-finding algorithm is programmed using MATLAB language and conducted for these grayscale images. The threshold selection in this algorithm can directly influence the accuracy of edge analysis. In this research, the edge exaction can be implemented exactly when the threshold is 0.02, as shown in Fig. 7b. It is shown that the boundary between the uncamouflage lens and its background is quiet distinct. As for camouflage lens, it is hard to distinguish.

By changing the designs of the microfluidic channels on the lens, glasses with various camouflage effects can be easily realized to adapt to other different backgrounds. Compared with complex camouflage technology (Kang et al. 2015; Yu et al. 2014), this liquid colour-changing camouflage technology by microfluidic channels shows the advantages of simple principle, easy realization and good performances. The camouflage PDMS film can also be applied for other surfaces of human body or machines.

### Optical filter

**Fig. 8:**
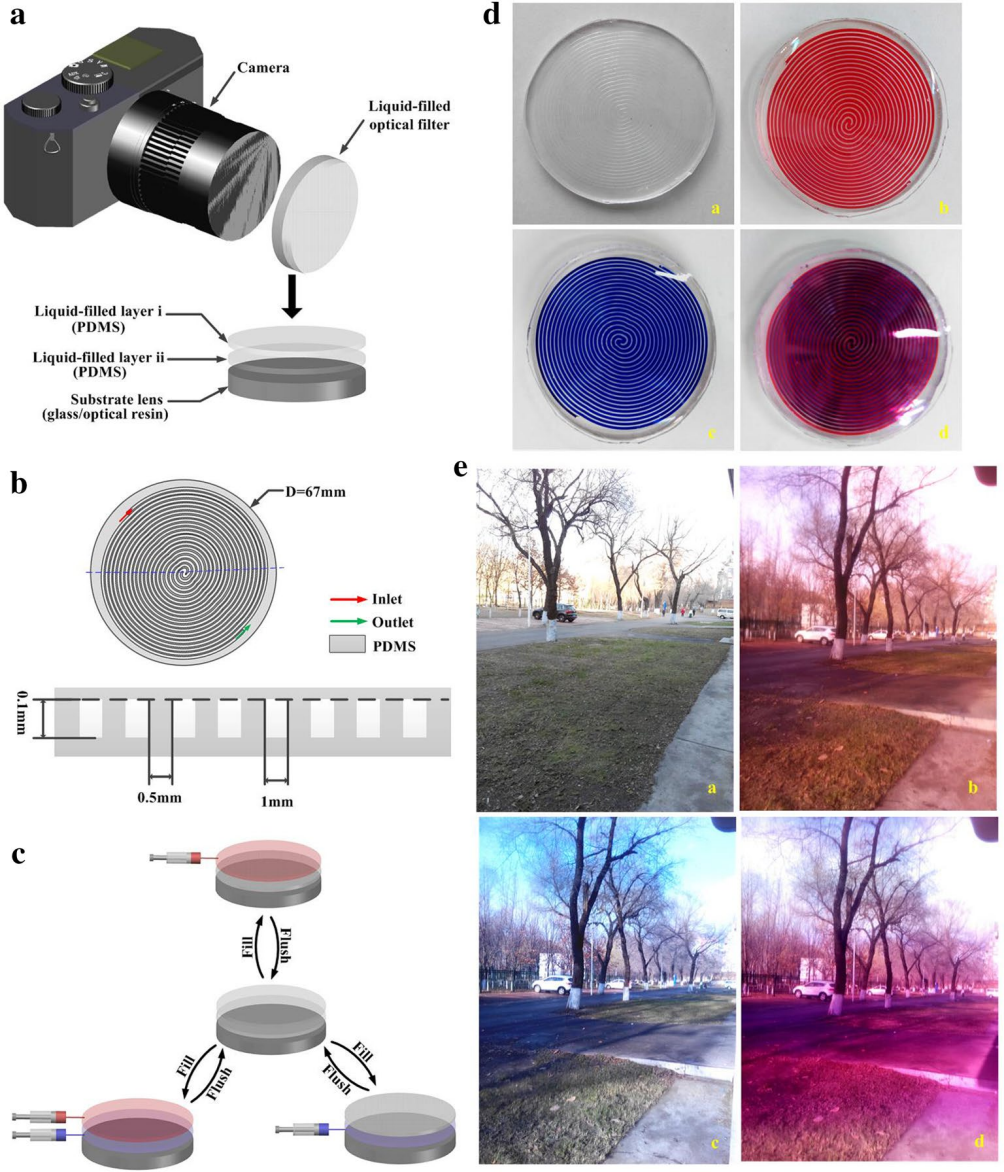
Liquid optical filter. **a** Structural schematic diagram. **b** Designs of the microfluidic channels on the optical filter (*top*) and cross-sectional schematic of the region indicated by the *dotted blue line* (*bottom*). **c** Colouration ways of the liquid-filed optical filter with two layers. **d** Images of the fabricated optical filter under different colouration ways: **a** without colouration, **b** fill red liquid in colour-changing layer i, **c** fill blue liquid in colour-changing layer ii, **d** fill red and blue liquids in colour-changing layer i and layer ii respectively. **e** Optical filtered pictures taken with this optical filter under different colouration ways in Fig. 7d (**a**–**d**)

An optical filter based on the liquid colour-changing lens with double layers is designed and fabricated as shown in Fig. 8. It mainly consists of two PDMS liquid colour-changing layers and a glass/optical resin (CR39) substrate lens (Fig. 8a). The width and interval of the channels on the lens are 1 and 0.5 mm respectively, the depth is made to be 0.1 mm (Fig. 8b). The filter colours can be changed by circulating different colour liquids into the channels on different layers manually or automatically according to different photographic needs (Fig. 8c, d). Various photography effects are shown by using this optical filter (Fig. 8e). When the liquid colour-changing layer i of the filter is filled with red liquid, most of the blue and green light are absorbed and red light passes through leading to a bright red tone. When liquid colour-changing layer ii is filled with blue liquid, most of the yellow light are absorbed and blue light can get through resulting in a blue tone. And if the two liquid colour-changing layers are filled with red and blue liquids respectively, a purple tone is obtained.

More photography effects can be obtained by this liquid colour-changing optical filter through changing the colours liquids filled with. Three or more layers of channels can also be easily fabricated for overlap effect. Compared with the conventional coated optical filter, this liquid colour-changing optical filter shows more flexible function, easier making and better colour uniformity.

In addition to above applications, some other designs of the channels and applications of the liquid colour-changing lenses can also be implemented. For example, when applied on the rearview mirror of cars, they can decrease the intensity of the reflected light, reduce glare effects and comfort driver’s eyes by the colour liquids filled with. When applied on windows of cars, airplanes or rooms, they can reduce the transmitting intensity of light and prevent human privacy automatically or under one’s control.

## Conclusions

From the designs and fabrication of the liquid colour-changing lenses with microfluidic channels above, it can be seen that the microfluidic liquid colour-changing lenses with different designs can be easily and quickly fabricated by soft lithography technology with proper fabrication parameters. New surface treatment method for the irreversible bonding of PDMS with CR39 was investigated and proved to be effective. Carefully designed liquid colour-changing lenses can provide vision protection, camouflage and optical filtering functions for human. In addition to the successful implementation of their fundamental functions, the distinct advantages of the lenses for these applications over previous ways are the simplicity in manufacturing, easy operating and good controllability. Although we have focused on optometry field, other designs and applications of these microfluidic liquid colour-changing systems interfaced with other devices are proposed for presenting new opportunities for modifying their appearance.

## References

[CR1] Armistead WH, Stookey SD (1964). Photochromic silicate glasses sensitized by silver halides. Science.

[CR2] Becker H, Gaertner C (2008). Polymer microfabrication technologies for microfluidic systems. Anal Bioanal Chem.

[CR3] Chang ST, Ucar AB, Swindlehurst GR, Bradley RO, Renk FJ, Velev OD (2009). Materials of controlled shape and stiffness with photocurable microfluidic endoskeleton. Adv Mater.

[CR4] Dimitrova M, Merilaita S (2014). Hide and seek: properties of prey and background patterns affect prey detection by blue tits. Behav Ecol.

[CR5] Duffy DC, McDonald JC, Schueller OJA, Whitesides GM (1998). Rapid prototyping of microfluidic systems in poly (dimethylsiloxane). Anal Chem.

[CR6] Eddings Mark A, Johnson Michael A, Gale Bruce K (2008). Determining the optimal PDMS–PDMS bonding technique for microfluidic devices. J Micromech Microeng.

[CR7] Fuentes-Fernandez J, Cuevas S, Alvarez-Nunez LC, Watson A (2013). Tests and evaluation of a variable focus liquid lens for curvature wavefront sensors in astronomy. Appl Opt.

[CR8] Hemmil S, Cauich-Rodríguezc JV, Kreutzer J, Kallio P (2012). Rapid, simple, and cost-effective treatments to achieve long-term hydrophilic PDMS surfaces. Appl Surf Sci.

[CR9] Iimura Y, Onoe H, Teshima T, Heo YJ, Yoshida S, Morimoto Y, Takeuchi S (2015). Liquid-filled tunable lenticular lens. J Micromech Microeng.

[CR10] Kang C, Stevens M, Moon JY, Lee SI, Jablonski PG (2015). Camouflage through behavior in moths: the role of background matching and disruptive coloration. Behav Ecol.

[CR11] Lee SH, Rhee HW, van Noort D, Lee HJ, Park HH, Shin IS, Hong JI, Park TH (2014). Microfluidic bead-based sensing platform for monitoring kinase activity. Biosens Bioelectron.

[CR12] Liberale C, Cojoc D, Bragheri F, Minzioni P, Perozziello G, La Rocca R, Ferrara L (2013). Integrated microfluidic device for single-cell trapping and spectroscopy. Sci Rep.

[CR13] Lim JL, Hu DJJ, Shum PP, Wang YX (2014). Design and analysis of microfluidic optical fiber device for refractive index sensing. IEEE Photon Technol Lett.

[CR14] Liu P, Mao DP, Martin RJ, Dong L (2012). An integrated fiber-optic microfluidic device for detection of muscular force generation of microscopic nematodes. Lab Chip.

[CR15] Majedi FS, Hasani-Sadrabadi MM, Emami SH, Shokrgozar MA, VanDersarl JJ, Dashtimoghadam E, Bertsch A, Renaud P (2013). Microfluidic assisted self-assembly of chitosan based nanoparticles as drug delivery agents. Lab Chip.

[CR16] McDonald JC, Whitesides GM (2002). Poly (dimethylsiloxane) as a material for fabricating microfluidic devices. Acc Chem Res.

[CR17] McDonald JC, Duffy DC, Anderson JR, Chiu DT, Wu HK, Schueller OJA, Whitesides GM (2000). Fabrication of microfluidic system poly (dimethylsiloxane). Electrophoresis.

[CR18] Moon KS, Choi JH, Choi DJ, Kim SH, Ha MH, Mam HJ, Kim MS (2008). A new method for analyzing the refill process and fabrication of a piezoelectric inkjet printing head for LCD color filter manufacturing. J Micromech Microeng.

[CR19] Morin SA, Shepherd RF, Kwok SW, Stokes AA, Nemiroski A, Whitesides GM (2012). Camouflage and display for soft machines. Science.

[CR20] Ng Alphonsus HC, Uvaraj Uddayasankar, Wheeler Aaron R (2010). Immunoassays in microfluidic systems. Anal Bioanal Chem.

[CR21] Paul Y, Edwards T, Fu E, Helton K, Nelson K, Tam MR, Weigl BH (2006). Microfluidic diagnostic technologies for global public health. Nature.

[CR22] Qin D, Xia YN, Whitesides GM (1996). Rapid prototyping of complex structures with feature sizes larger than 20 μm. Adv Mater.

[CR23] Ren H, Wu ST (2005). Variable-focus liquid lens by changing aperture. Appl Phys Lett.

[CR24] Roy A, Ghatak A (2014). Design of an adaptable optofluidic aspherical lens by using the elastocapillary effect. Adv Opt Mater.

[CR25] Santiago-Alvarado A, Gonzalez-Garcia J, Itubide-Jimenez F, Campos-Garcia M, Cruz-Martinez VM, Rafferty P (2013). Simulating the functioning of variable focus length liquid-filled lenses using the finite element method (FEM). Optik.

[CR26] Shih SCC, Gach PC, Sustarich J, Simonss BA, Adama PD, Singh S, Singh AK (2015). A droplet-to-digital (D2D) microfluidic device for single cell assays. Lab Chip.

[CR27] Surmacki A, Ozarowska-Nowicka A, Rosin ZM (2013). Color polymorphism in a land snail Cepaeanemoralis (Pulmonata: Helicidae) as viewed by potential avian predators. Naturwissenschaften.

[CR28] Taichung TW, Hsinchu TW (2004) Method for fabricating color filter. US Patent 6,830,856, 14 DEC 2004

[CR29] Thuillier G, Malek CK (2005). Development of a low cost hybrid Si/PDMS multi-layered pneumatic microvalve. Microsyst Technol.

[CR30] Tian BZ, Zhang JL (2012). Morphology-controlled synthesis and applications of silver halide photocataly materials. Catal Surv Asia.

[CR31] Tseng WY, Fisher JS, Prieto JL, Rinaldi K, Alapati G, Lee AP (2009). A slow-adapting microfluidic-based tactile sensor. J Micromech Microeng.

[CR32] Watson AC, Siemann LA, Hanlon RT (2014). Dynamic camouflage by Nassau groupers *Epinephelus striatus* on a caribbean coral reef. J Fish Biol.

[CR33] Yang C, Shen WD, Zhang YG, Li K, Fang X, Zhang X, Liu X (2015). Compact multilayer film structure for angle insensitive color filtering. Sci Rep.

[CR34] Yoon YT, Lee SS (2010). Transmission type color filter incorporating a silver film based etalon. Opt Express.

[CR35] Yu CJ, Li YH, Zhang X, Huang X, Malyarchuk V, Wang SD, Shi Y, Gao L, Su YW, Zhang YH (2014). Adaptive optoelectronic camouflage systems with designs inspired by cephalopod skins. Proc Nati Acad Sci USA.

[CR36] Zhao PP, Ataman C, Zappe H (2015). Spherical aberration free liquid-filled tunable lens with variable thickness membrane. Opt Express.

